# The Cultural Adaptation of Step-by-Step: An Intervention to Address Depression Among Chinese Young Adults

**DOI:** 10.3389/fpsyt.2020.00650

**Published:** 2020-07-07

**Authors:** Hao Fong Sit, Rui Ling, Agnes Iok Fong Lam, Wen Chen, Carl A. Latkin, Brian J. Hall

**Affiliations:** ^1^Global and Community Mental Health Research Group, Department of Psychology, University of Macau, Macau, China; ^2^Department of International Health, Johns Hopkins Bloomberg School of Public Health, Johns Hopkins University, Baltimore, MD, United States; ^3^Department of Communication, Faculty of Social Science, University of Macau, Macau, China; ^4^Centre for Macau Studies, University of Macau, Macau, China; ^5^Department of Medical Statistics, School of Public Health, Sun Yat-sen University, Guangzhou, China; ^6^Health, Behavior, and Society, Johns Hopkins Bloomberg School of Public Health, Baltimore, MD, United States

**Keywords:** digital mental health, cultural adaptation, depression, intervention, young adults

## Abstract

**Background:**

Digital mental health interventions leverage digital communication technology to address the mental health needs of populations. Culturally adapting interventions can lead to a successful, scalable mental health intervention implementation, and cultural adaptation of digital mental health interventions is a critical component to implementing interventions at scale within contexts where mental health services are not well supported.

**Objective:**

The study aims to describe the cultural adaptation of a digital mental health intervention Step-by-Step in order to address depression among Chinese young adults.

**Methods:**

Cultural adaptation was carried out in four phases following Ecological Validity Model: (1) stage setting and expert consultation; (2) preliminary content adaptation; (3) iterative content adaptation with community members; (4) finalized adaptation with community feedback meetings. Cognitive interviewing was applied to probe for relevance, acceptability, comprehensibility, and completeness of illustrations and text. Six mental health experts and 34 Chinese young adults were recruited for key informant interviews and focus group discussions.

**Results:**

We adapted the text and illustrations to fit the culture among Chinese young adults. Eight elements of the intervention were chosen as the targets of cultural adaptation (e.g., language, metaphors, content). Samples of major adaptations included: adding scenarios related to university life (*relevance*), changing leading characters from a physician to a peer and a cartoon (*acceptability*), incorporating two language versions (traditional Chinese and simplified Chinese) in the intervention (*comprehensibility*), and maintaining fundamental therapeutic components (*completeness*).

**Conclusion:**

This study showed the utility of using Ecological Validity Model and a four-point procedure framework for cultural adaptation and achieved a culturally appropriate version of the Step-by-Step program for Chinese young adults.

## Introduction

Digital mental health interventions leverage digital communication technology to address the mental health needs of populations. These interventions incorporate information provision, screening, assessment, and monitoring, intervention, and social support (1). Key advantages of using this innovative technology include reductions in treatment barriers such as time and geographical boundaries, and treatment costs while offering timely responses to mental health needs (2, 3). A meta-analysis of 17 trials of 1,480 participants showed that computerized and web-based intervention were effective at reducing depression and anxiety compared with inactive control (4). A systematic review of 89 studies demonstrated that nearly 81% of digital mental health programs are effective or partially effective at reducing a variety of symptoms of common mental disorders and improving well-being (5).

In response to the global need for scalable mental health interventions, the World Health Organization developed Step-by-Step (SbS), a transdiagnostic and guided digital mental health intervention primarily targeting symptoms of common mental disorders, including depression (6–10). The program utilizes narrative stories with illustrations to teach participants behavioral activation techniques and skills (e.g., activity scheduling), provide information (e.g., list for social and health service providers), augmented with nonspecialist guidance (10). It consists of five 20–30 minutes sessions that teach techniques of stress management, planning physical and social activities, reducing avoidance coping, improving self-acceptance, and preventing relapse. The narrative stories involved a character (service recipient, the junior leading character) who learned how to better cope with daily stressors in different scenarios (e.g., family conflicts, feeling isolated) and manage their mood based on knowledge imparted by a doctor (service provider, the senior leading character). Behavioral activation, as the core therapeutic technique, gets participants active in daily life by scheduling activities to enhance their mood and are effective in managing depression symptoms (11). In sessions of SbS, lists of activities are offered for users who can choose from these activities or come up with their own. Also, a list of social and health service providers was provided to increase social interaction by seeking help from others. The intervention content, guidance model, and mobile application are three essential components of the program (7), which can be adapted for various cultural groups. SbS has been adapted for Filipino migrant workers (8), and Syrian refugees, Lebanese, and Palestinians in Lebanon (12) through a cultural adaption process involving community engagement with key local community members and stakeholders.

Culturally adapting interventions is one of the keys to successful scalable mental health intervention implementation (13). Cultural adaptation is “the systematic modification of an evidence-based treatment (or intervention protocol) to consider language, cultural, and context in such a way that it is compatible with the client’s cultural patterns, meaning, and values” (14). Incorporating cultural elements, including spiritual beliefs and social norms, and take account of the local context and needs of the target population can improve service users’ attitudes toward the intervention (15). Several studies have highlighted considerations for incorporating culture in the implementation of mental health interventions in low-and-middle-income countries (16–19), but these studies focus on nontechnology-supported intervention. Relatively few cultural adaptation studies for digital mental health interventions have been conducted within this context, leaving a critical gap in the literature. Abi Ramia and colleagues (12) culturally adapted a digital mental health intervention in Lebanon through learning participants’ receptiveness to the program, its content, implementation, and prospective use with local communities and stakeholders. Garabiles and colleagues (8) completed a cultural adaptation to enhance the acceptability, relevance, comprehensibility of the intervention and its content (e.g., storyline, illustration, and characters) while maintaining the completeness of the therapeutic components. Previous work adopted a framework for guidance on what elements should be adapted (20) and rationale behind adaptations (8) to ensure the thoroughness of the cultural adaptation within the digital format. Digital mental health intervention adaption must meet community needs without the reliance on explanations and modifications by human supports (8, 21), which highlights the importance of creating intervention material that can stand alone.

Several models are known for cultural adaptation with distinguishing features (22), such as tailoring for specific ethnic groups (23, 24), therapeutic process and skills (25), elements within the intervention (26), specific domains that have a poor fit between the intervention and the community (27), and heuristic considerations including patient engagement, mechanism, and treatment outcomes (28). Frameworks applied for cultural adaptation of digital mental health intervention include Ecological Validity Model (26), Heuristic Framework (28), Integrative Cultural Adaption Model (29), Formative Method for Adapting Psychotherapy (30), and four-point Framework (31), and all of which were designed to inform the adaptation process except the Ecological Validity Model (EVM) (26). The Ecological Validity Model addressed eight culturally sensitive domains within an intervention, which are language (i.e., culturally appropriate and syntonic language), people (i.e., ethnic/racial similarities), metaphor (i.e., common symbols and concepts shared within the cultural group), content (e.g., cultural knowledge about values, customs, and traditions), concepts (e.g., culturally and contextually consonant treatment constructs), goals (i.e., formation of an agreement on the treatment goal), methods (i.e., procedures/model used to accomplish the treatment goal), and context (i.e., psychological, developmental, social, economic and political context) (32). Using the Ecological Validity Model (26), eight culturally sensitive dimensions for intervention were explored with mental health providers to culturally adapt a web-based depression intervention for Latino adolescents (20). This study also highlighted that it is important to learn from both providers/experts and users’ perspectives to enhance the applicability of the program and also maintain the therapeutic quality of the intervention. Arjadi and colleagues (33) applied the Ecological Validity Model to adapt an internet-based behavioral activation intervention addressing depression for Indonesian people. In addition, an internet-based intervention for depression was culturally adapted for Colombian university students using this model (34) and was evaluated (35). Evidence from a meta-analysis of eight randomized controlled trials showed that the effectiveness of minimally guided digital mental health interventions to reduce symptoms of common mental disorders was enhanced by cultural adaptation (36). Cultural adaptation of digital mental health interventions is a vital component to implementing interventions at scale within contexts, especially where mental health services are not well supported.

Frameworks for cultural adaptation are generally used to inform adaptation dimensions for intervention content and to inform the process of the adaptation (37). The current study adopted the EVM (26) to inform which intervention elements should be culturally adapted. The framework, including eight culturally sensitive dimensions (i.e., language, persons, metaphors, content, concepts, goals, methods, context) was previously applied in cultural adaptation studies (20, 22, 34). For the framework informing the strategies and process of cultural adaptation, the current study did not follow any existing cultural adaptation process model (22, 37, 38) since they were not appropriate for digital mental health program as digital mental health interventions minimize contact between the providers and service users. The main focus of the adaptation study was on the intervention content (e.g., text, storyline, and illustrations), which comprise the self-guided information received by the service users. A process model with four phases was applied to emphasize the input provided by representatives of the intervention target population and the adaptation of the materials (8).

## Methods

We selected the EVM model developed by Bernal and colleagues (26) to inform the domains of the content modifications to ensure language, persons, metaphors, content, concepts, goals, methods, and contexts in this version of the SbS intervention were acceptable, relevant, comprehensible, and complete for Chinese young adults.

### Participants and Data Collection

Purposeful sampling was used to invite six Chinese mental health experts from Macao and mainland China for key informant interviews (KIIs). All of them earned advanced postgraduate degrees in mental health fields, including counseling, clinical psychology, and psychiatry and had extensive experience working with Chinese clients. We balanced the sampling to represent both Cantonese speaking experts and Mandarin speaking experts since our aim in the adaptation was to develop programs for these two distinct linguistic and cultural groups. All interviews lasted for 90 to 120 minutes and were audio recorded with consent from participants.

Chinese young adults who were 18 years or older and lived in Macao for at least one year were eligible to participate in the focus groups (FGDs). Participants were recruited through purposive sampling in cooperation with the General Association of Chinese Students of Macao, the largest NGO that works for Chinese students in Macao. We composed four homogenous groups based on sex (female or male) and origin of birth (Macao Chinese or mainland Chinese) to encourage all participants to speak freely among peers. We conducted 18 FGDs, five each with Macao Chinese women and men, and four each with mainland Chinese women and men. The average number of participants in Macao male group, Macao female group, mainland male group, and mainland female group are 6 (6–7), 6 (5–8), 5 (4–6), and 7 (5–8). There were 41 participants in total, and each FGD lasted about 3 hours. All FGDs were recorded with written consent. The FGDs were open groups, with not all participants attending all sessions. In order to maintain a group size of about eight participants, new participants were recruited by snowball sampling throughout the study.

**Figure 1:**
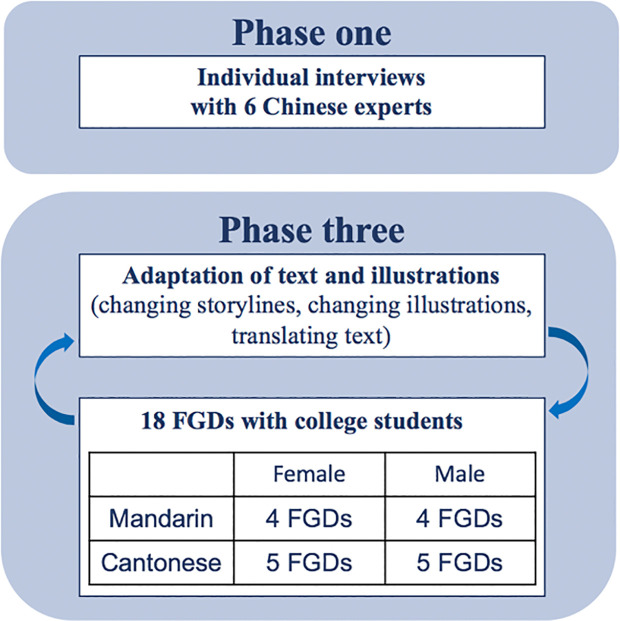
Data collection process.

Basic demographic information (age, sex, marital status, highest education level, whether they were born in Macao, number of years living in Macao, and job experience in Macao) was collected before KIIs and FGD, and in addition, data on depressive (39) and anxiety symptoms (40), and wellbeing (41) was collected from FGD participants. Data was obtained through online surveys using Qualtrics (**Figure 1**).

### Procedures

**Figure 2:**
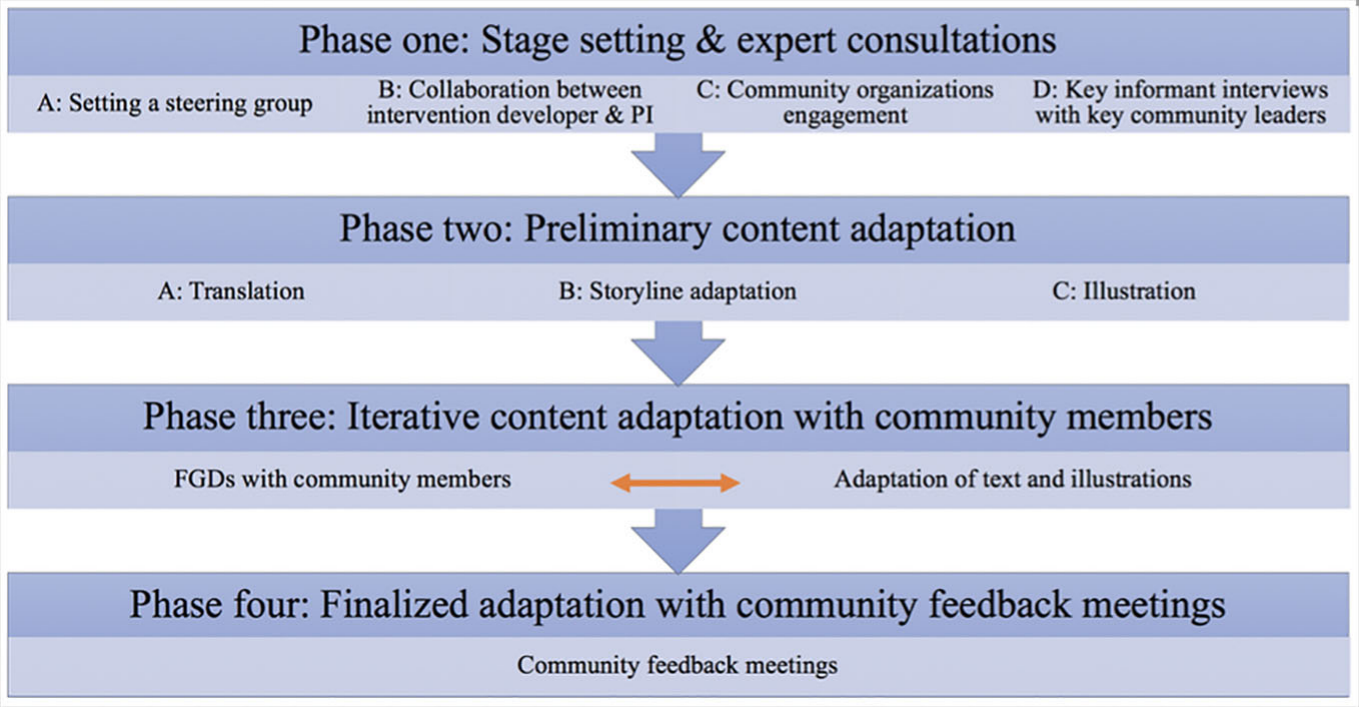
Cultural adaptation process.

The study was conducted in four phases from May 2019 to December 2019 (see **Figure 2**). The phases were: (1) expert consultations; (2) preliminary content adaptation for the intervention materials; (3) iterative content adaptation with community members; (4) finalized adaptation with community feedback meetings.

#### Phase One: Stage Setting and Expert Consultations

Community engagement is important for cultural adaptation (22). In our study, stakeholders, including a WHO technical officer, mental health experts, and local community groups, were involved in different phases. In phase one, we set up a steering group to collaborate with stakeholders and implement the study. The steering group included the project principal investigator (PI), a professor in clinical psychology and public health who previously completed another cultural adaption of the intervention with a different population (8), a professor in communication (co-PI), and two Chinese graduate students, who had a clinical psychology, anthropology, and public health background, and who were responsible for data collection and analysis. As the intervention developer, WHO provided technical support and advice for the study. The steering group also built upon an existing and collaborative relationship with the Student Affairs Office (SAO) of the University of Macau and the General Association of Chinese Students of Macao (AECM) to support the study.

The last step of Phase One was the conduct of six semistructured, KIIs with mental health experts from May to June to understand the mental health context of Chinese young adults. Before the interviews, the mental health experts independently reviewed the SbS program material to ensure the efficiency of the interviews. During the interview, the interviewer (a graduate student in the steering group) started the conversation with a welcome opening, the introduction of the program and the purpose of the interview, and oral informed consent was obtained. After starting the recording, the interviewer began with an open question about their experience with Chinese clients in general, followed by their professional perspectives on specific age groups and about the mental health context in Macao. After questions about major mental health problems, existing mental health services, and client’s typical coping strategies, we focused on the content of SbS with questions related to the appropriateness of the program, delivery method, characters, storyline, illustrations, and expressions. The notetaker (the other graduate student in the steering group) organized the notes after the interview.

#### Phase Two: Preliminary Content Adaptation for the Intervention Materials

The content of the original version of SbS was written in English. In phase two, a team member with a minor in translation studies translated the English language into the Chinese language, and a different team member who was bilingual (English & Chinese) conducted backward translation to check the accuracy of the translation.

We recruited a creative story writer with a background in Chinese education to change the storyline for Chinese students based on the feedback in Phase One from KIIs with the mental health experts. We also hired a local professional illustrator with a background in visual art & art education to design the main characters and the illustrations in session one for future discussion. A simplified Chinese version was adapted from the traditional Chinese version to ensure that both Macao Chinese and mainland Chinese could utilize the program materials.

#### Phase Three: Iterative Content Adaptation With Community Members

For phase three, the study team worked in conjunction with AECM, who was responsible for participant recruitment. We had a Mandarin working group with one facilitator and three note-takers who spoke Mandarin as their first language as well as a Cantonese working group with one facilitator and two note-takers who are Cantonese speakers. They were all trained in properly organizing focus groups and in avoiding leading questions or intimidating questions by the PI and co-PI. The note-takers also prepared PowerPoints with all materials in one session before the FGDs, which would be shown to the participants during the FGDs. The main purpose of the FGD was to adapt the storyline and to apply a bottom-up approach to generate the illustrations with participants, meaning that participants would provide ideas for the illustrations based on the texts and discussion, and then the illustrator would develop the illustrations based on ideas generated from the FGDs. Illustration adaptation and FGDs discussions were iterative and concluded when FGD participants were satisfied with the illustrations.

All focus groups were conducted in a private meeting room with a projector at a service center of AECM. The working groups were matched with participants by language. During the FGD, the facilitator presented the program introduction, the purpose of the study, and basic rules for the discussion and started the discussion with participants’ short self-introduction as an ice-breaking activity. After that, facilitators focused on the materials using cognitive interviewing strategies (8). Participants were asked to describe what they thought was occurring in the intervention text, and follow-up questions were used to guide their comments on how the text and illustrations should be changed or developed. Detailed interview guide for focus group discussion is presented in **Supplement 1**.

#### Phase Four: Finalized Adaptation With Community Feedback Meetings

For phase four, we had two more community meetings with the local students who participated in the FGDs when we presented the new illustrations and suggested changes from the results of FGDs. Feedback was collected and analyzed to inform the further changes. Adaptation decisions were finalized during the community feedback meeting. Finally, the adaptation results were documented according to the eight elements of the EVM (26).

### Analysis

The notetaker of the KIIs took detailed notes and organized the notes with audio recordings during and after the KIIs. The notes were synthesized by the notetaker and the interviewer using excel. The main thematic codes were Chinese mental health, cultural considerations, potential target tailoring groups, and the SbS digital intervention. The eight elements of the EVM framework were used as sub-theme codes under SbS digital intervention to further synthesize the adaptation of materials of the intervention.

Two working groups led by the graduate students in the steering groups took detailed notes during all FGDs. Suggestions for changes of text & storyline, the ideas for illustrations, and rationale for changes were recorded. After the FGDs, we organized steering group meetings to discuss the suggestions to ensure they were clear and consistent with the theorized treatment mechanism of symptom improvement. The results of the FGDs and steering group meetings were coded using excel through the lens of the Bernal’s EVM model (26). The results of all potential changes were presented in the community feedback meetings in the last phase to finalize the adaptation.

### IRB Approval

IRB approval was granted by the University of Macau.

## Results

### Participants’ Characteristics

A total of 41 Chinese young adults participated in the FGD (26 Macao Chinese). Among local students (19.84 yrs, 18–25), ten of them are male, and 23 were born in Macao. Among nonlocal students (19.26 yrs, 18–21), seven of them of male. Descriptive statistics and means and standard deviations of depression, anxiety, and subjective well-being are shown in **Table 1**.

**Table 1:** Sociodemographic and clinical characteristic.

	Macao Chinese (N=26)			Mainland Chinese (N=15)		
	M	SD	N (%)/range	M	SD	N (%)/range
Age	19.8	1.5	18-25	19.3	0.7	28-21
Gender						
Male			10 (38.5%)			7 (46.7%)
Female			16 (61.5%)			8 (53.3%)
Marital status (unmarried)			26 (100%)			15 (100%)
Born-in-Macao			23 (88.5%)			1 (93.3%)
Number of years living in Macau	18.5	3.5	7-25	1.6	1.12	1-5
PHQ-9^a^	6.5	4.5		5.4	3.1	
PHQ-9 ≥ 10			6 (23.1%)			2 (13.3%)
GAD-7^b^	4.8	3.7		3.6	3.4	
GAD-7 ≥ 10			3 (11.5%)			1 (6.7%)
WHO-5^c^	10.8	5.3		9.7	6.0	
WHO-5 < 13			16 (61.5%)			10 (66.7%)

a PHQ-9, Patient Health Questionnaire-9; a score equal or above 10 indicates depression.

b GAD-7, General Anxiety Disorder-7; a score equal or above 10 indicates anxiety.

c WHO-5, World Health Organization–Five Well-Being Index; a score below 13 indicates poor well-being as well as an indication for testing for depression under ICD-10.

### Cultural Adaptation

Eight dimensions within the EVM (26) were applied for cultural adaptation of Chinese SbS, which are language, people, metaphor, content, concepts, goals, methods, and context to enhance relevance, acceptability, and comprehensibility while maintaining the completeness of therapeutic elements of the intervention.

#### Language

Several issues related to comprehensibility and acceptability were identified in terms of language during FGDs. In terms of comprehensibility, several FGDs pointed out that the translations were redundant across sessions. This was improved by either combining similar expressions or skipping redundant content while maintaining the coherence of the storylines and therapeutically meaningful components. Examples of changes are shown in **Table 2**.

**Table 2:** Samples of adaptation.

	Example (before adaptation)	Issue	Adaptation decision	Example (after adaptation)
**Language**	“And a warm welcome to Session 1. It’s great to see you back here, ready for this next step.”	Comprehensibility: the expression was redundant	combined the expression and make it less redundant and clearer.	“We welcome you to come back to Session 1, and we can prepare for next step now.”
	“special day”	Comprehensibility: The expression was hard to understand in the context	used local idiom/phrase	“big day”
	“leave my worries in the car”			“throw it behind the brain”
	“Well done!”	Acceptability: the encouragement messages appeared very often but Chinese usually refrain from expressing compliments verbally	changed word into image	**Figure 3**
	“my dear” (“Habibti” in Arabic version)	Acceptability: “My dear” is not a common Chinese expression, and Chinese young adults seldom use “my dear” to their close friend or their important one.	change it to character’s name for the local Chinese young adults and nonlocal young men.	character’s name
**People**	medical doctor or psychiatrist as the senior character profession	Acceptability: people who see a medical doctor or psychiatrist must have severe problems and need hospitalization or they believed that they could seek a more suitable professional to cope with their problems	the appearance of senior character did not imply medical doctor or psychiatrist	a cartoon character for male and a young female in causal wear
	human figure as senior characters in male version	Acceptability: reminded the participants’ unpleasant experience with their mother or authorities in school whereas the cartoon figure was more approachable and comfortable to accompany with	did not use human figure as senior leading character in male	a cartoon character
	kind mother character, middle-aged female professional, and a cartoon	Acceptability: They seldom seek help and share their experience with someone not at their age. They tended to seek help from their female peer or senior students (Shijie) they feel close to and they believed that female senior students had similar experience with other college students and had experienced more in life so that they could understand students’ troubles and concerns.	applied a young female figure as senior leading character in female version	a young female as senior leading character (**Figure 3**)
**Metaphor**	“help with housework”	Relevance: the story was not clearer and relevant enough to young adults’ life experience,	added metaphors suggested by FGDs	“help clean your room, it looks like a doghouse” (“doghouse” is a metaphor for an untidy room in Chinese and was commonly heard from a parent, according to the female groups.)
	“carrying the weight of the world on your shoulder”	Acceptability & comprehensibility: too abstract and too exaggerating	used plain expression, which is common in Chinese	“like a ton of invisible pressure falls on you” (無形的壓力)
	“I could have slept for a thousand years”	Acceptability & comprehensibility: too exaggerating and not used in daily conversation	changed to a plain expression contained similar meaning that reflected a person’ tiredness and depth of sleep.	“I could finally sleep well”
**Content (narrative)**	the scenario of falling behind in study	Relevance: dealing with a noncontributing teammate in a group project is common than academically falling behind	adjusted the scenario to free-rider issue	free-rider issue
	hire a cab to go out	Relevance: Bus is a common public transportation to young adults and going out by cab (used in Arabic version) is too well-off and not common in this population	adjusted the transportation to bus	take bus
	giving hug to friend	Relevance: not a common practice among Chinese young adults	adapted the scenario according to FGDs’ experience	gentle touch on shoulder
	spending time with family and picnic	Relevance: Picnics are not common activities in China and Macao. This is no culture of having a picnic with family among the young Chinese adults	adapted the scenario according to FGDs’ experience	ang out with friend in café or Cha Chaan Teng (a type of restaurant popular in Macau)
**Content (concepts)**	“headache”	Relevance: it is irrelevant to their experience	Modified the expression to fit young adults’ experience	“dizziness”
	“felt uncomfortable doing something for myself”	Comprehensibility: it is hard to understand	Modified the expression to fit young adults’ experience	“could not cheer myself up no matter what I did”
	“thoughts go really fast”	Comprehensibility: It sounds a positive reaction to FGDs and not a symptom	Modified the expression to fit young adults’ experience	“thoughts go very messy”
	“felt tired and ache across shoulders and neck”	Relevance: It is irrelevant to their experience	Modified the expression to fit young adults’ experience	“feeble limbs, stomachache and headache”

**Figure 3:**
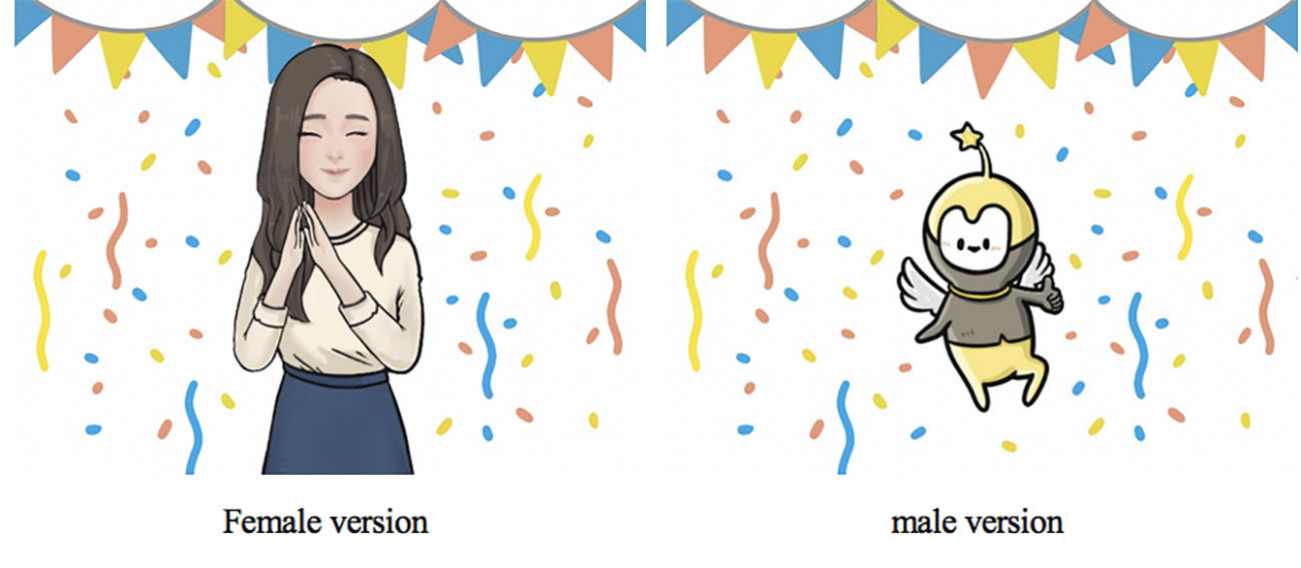
Senior leading characters (after adaptation); illustrations of expression of encouragement.

One major adaptation was made to increase the acceptability of the language. The use of encouragement and positive feedback such as “Well done!” were not seen as culturally consistent and were not acceptable. Participants reported that Chinese seldom express compliments verbally. Since these messages are important and designed to motivate users to continue the program, alternatives were needed. In order to enhance the acceptability of the use of encouragement and compliments, we generated illustrations that expressed encouragement, and modified messages to be acceptable if displayed with the illustration (**Figure 3**). The encouragement phrases were modified to explicitly emphasize the action they took and positive feedback, such as “you did a good job” and “you did very well.”

#### People

**Figure 4:**
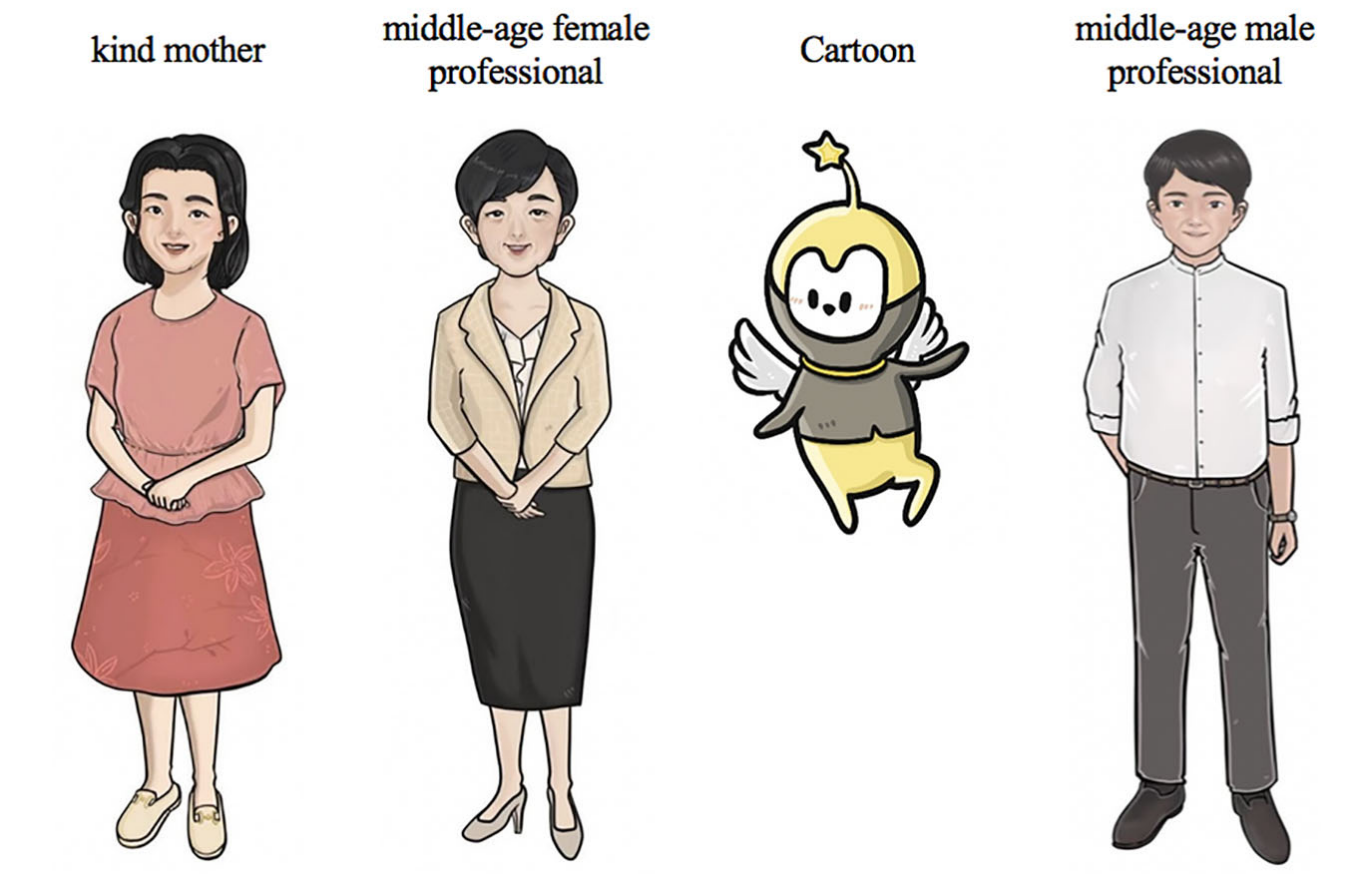
Senior leading characters (before adaptation).

A large difference between preferences for the service provider character between KIIs and FGDs was noted. The characters first suggested by the experts in KII, were a kind mother character, a middle-aged female professional, and a cartoon (see **Figure 4**). A middle-aged male professional character (see **Figure 4**) was added as requested by the local male group. We used a bottom-up approach that allowed participants to develop the characteristics of a character, including name, appearance, professional background. Both male groups considered that the human figures were not as acceptable as the cartoon character as a human figure reminded the participants’ unpleasant experience with their mother or authorities in school, whereas the cartoon figure was more approachable and comfortable to accompany with. Female groups preferred to talk with their peer or senior female students (*Shijie*). As a result, both male groups adopted a cartoon character, and both female groups adopted a young and experience female figure as the doctor figure in the original SbS to guide them through the program and to enhance the acceptability of SbS. Details of modification are shown in **Table 2**.

The cartoon figure was named “elf” in the local male version and “Xiao Q” in the nonlocal male version. The local female group picked the young female character (**Figure 3**), which was originally designed as the former service recipient, and named it Jessica. Details of adaptation for characters are shown in **Table 2**. Among local females, calling a friend or student by their nickname or English name is common, so we kept the English name for the senior leading character. The nonlocal female expressed that they tended to talk with a close girl-friend but was also willing to seek advice from a professional when they were upset. Thus, a young, reliable, female figure was used as the service provider character for the nonlocal female group. Similarly, they picked the young female character but maintained her identity Dr. Chen (Dr. as in PhD in psychology or counseling, not a medical doctor, expressed as *Boshi*, rather than *Yisheng*, in Chinese) to show her professionalism. The adapted and finalized senior leading characters are shown in **Figure 3**.

#### Metaphor

A few adaptations were made to improve the context where metaphors were used. According to KIIs, metaphor and cultural stories could be used when necessary but should be used cautiously as they might require knowledge of these stories to internalize the meaning. To make the story clearer and more relevant to young adults’ life experience, we added metaphors suggested by FGDs (**Table 2**). Some metaphors were thought to be too abstract and too exaggerated, and hence we made changes, such as “carrying the weight of the world on your shoulder” and “I could have slept for a thousand years”. We modified them according to the FGDs’ suggestion (**Table 2**).

#### Content

In the digital intervention SbS, content comprised narratives, characters, and illustrations. It contains specific cultural knowledge related to local values, customs, and traditions within a unique socioeconomic cultural background. Thus, content is a crucial part of this illustrated narrative intervention to enhance its cultural acceptability and relevance.

##### Narratives

The KIIs suggested that problems like family conflicts, addiction problems, and dating problems as relevant scenarios for young adults because they were more common issues in young adults. The storyline was modified according to KIIs’ feedbacks for FGDs. Male groups had very different feedback from the female groups. First, in terms of the background stressor of the junior leading characters, the male groups suggested “getting into a different university from their girlfriend’s and conflicts in romantic relationship,” they felt had strong relevance to the story. The female groups expressed that not every college student can relate to issues with their intimate partner, although dating is common in university. They suggested moving the stressor scenario as a romantic relationship in a later session and use “a family member diagnosed with a severe illness” as the primary trigger of the stress reaction. Second, in the original version, we included a scenario that narrated a friend came to the junior leading character’s home and talked with the character when the character was sad. Females reflected that that is what a close friend would do and agreed on the scenario, but the males reported that they seldom comforted a close friend by simply talking. Instead, they would first do something to help their friend distract from the unpleasantness, such as playing video games together. After a while, they would start to talk about the concerns. Thus, we adapted this scenario to include video game playing for the male versions.

**Figure 5:**
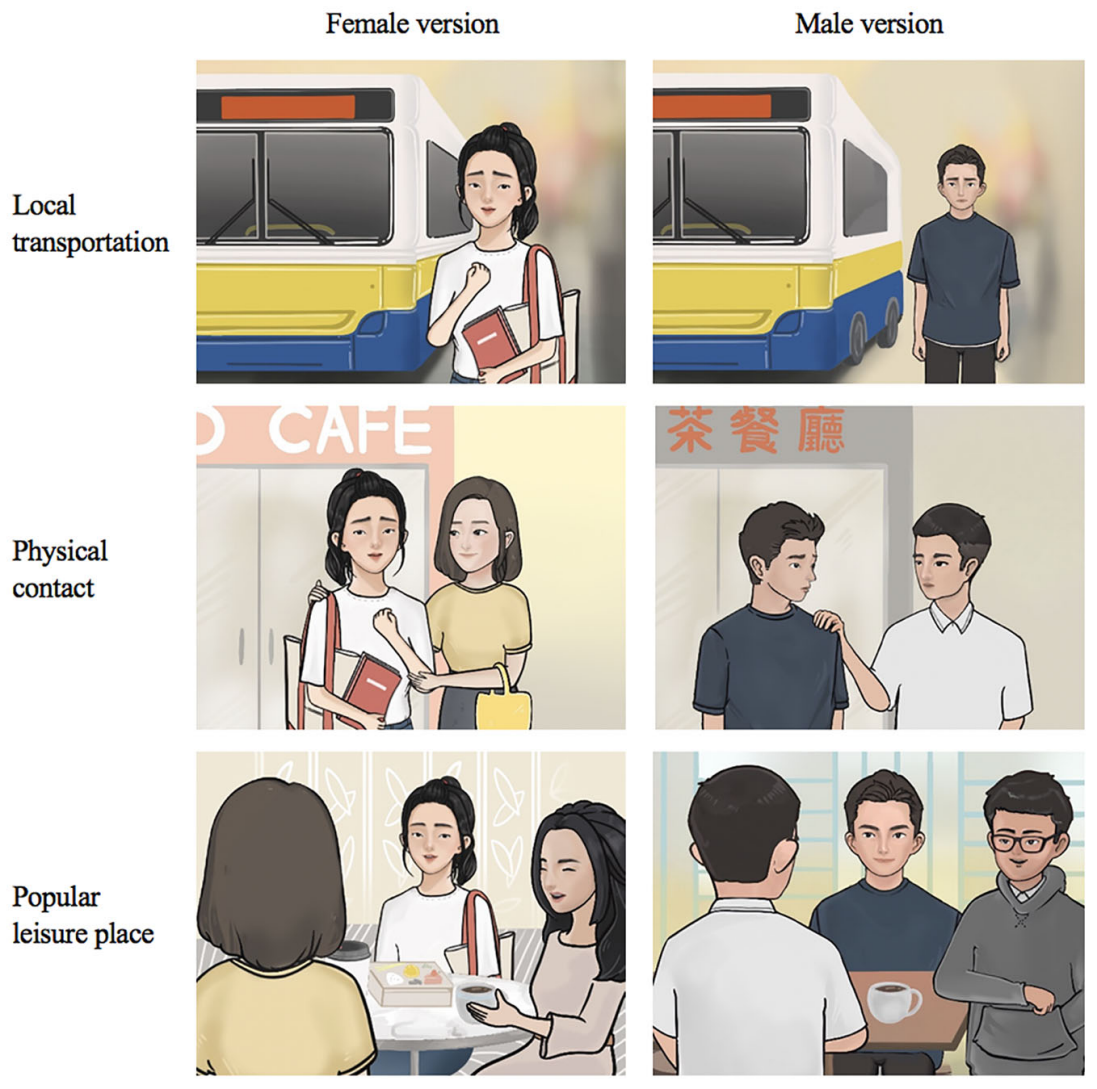
Examples of culture-related elements included in the content.

Both female and male groups shared similar challenges related to school work, such as problems in dealing with a noncontributing teammate in a group project (described as a “free-rider” in FGDs). Generally, all groups agreed that the free-rider issue was common to college students, and they felt it was hard to talk about the issue with the team member while not hurting their relationship. Thus, we included the free-rider issue in the story and modified it from our original idea, which was a worry about grades and falling behind in studies. We also adjusted the narratives by modifying some of the elements, such as transportation, interaction with friends, and places (see **Figure 5**).

##### Characters

**Figure 6:**
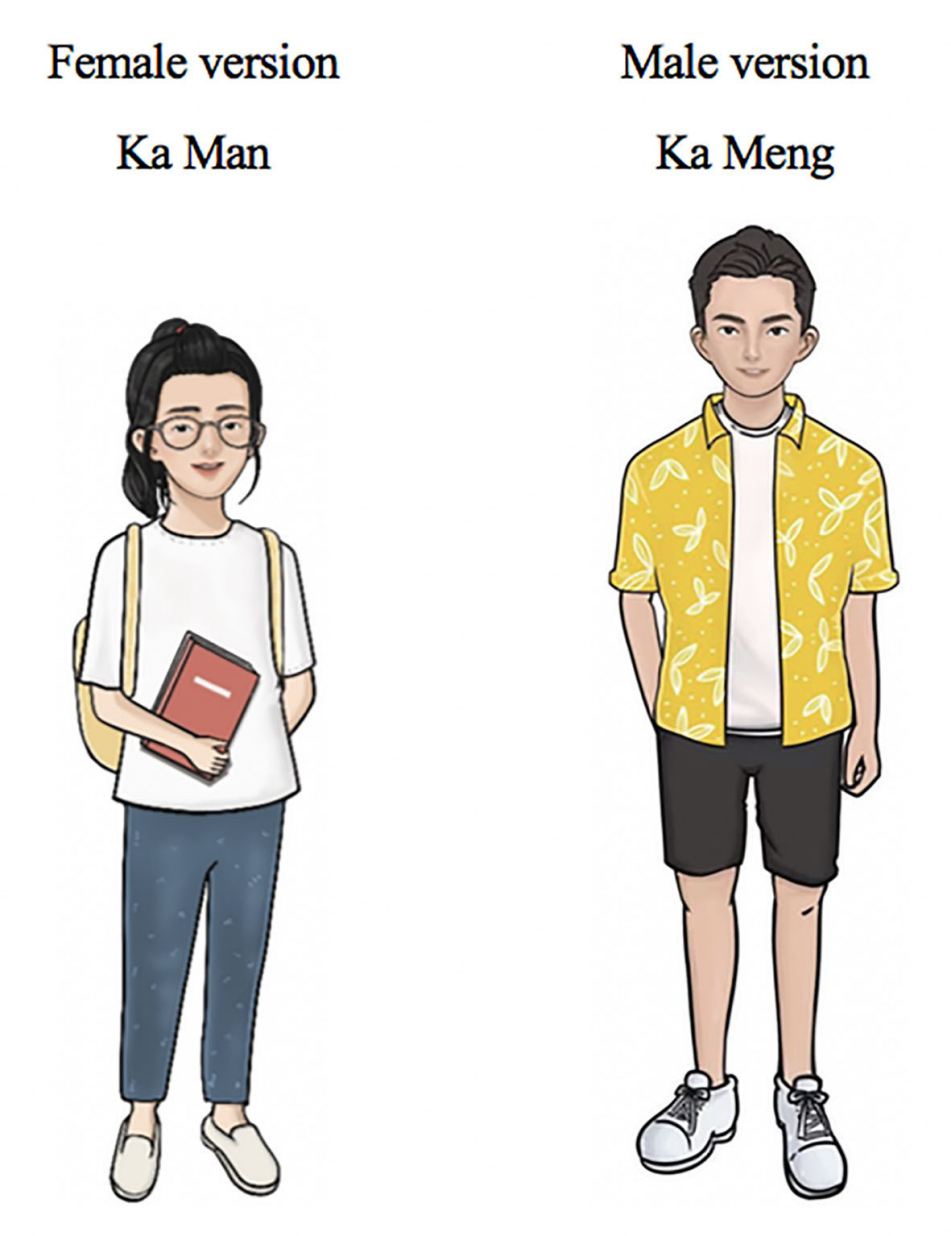
Junior leading characters (former service recipients).

**Figure 7:**
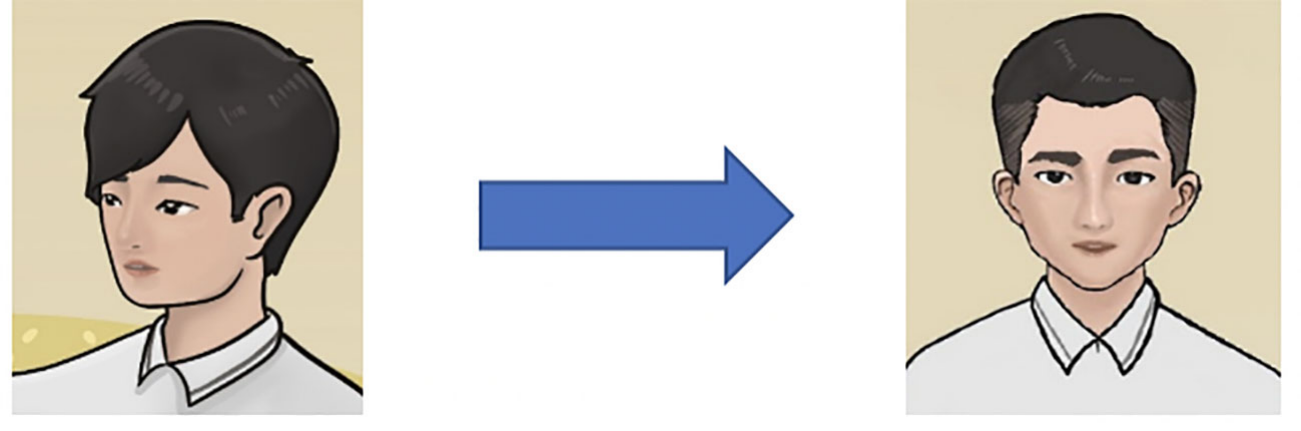
Adaptation of a peer character.

The junior leading characters (the former service recipients) were created by following several criteria: matching the target population’s gender, age, and style of dress (**Figure 6**). They were given a common Chinese name. The female groups preferred the female junior leading character to be younger and innocent compared to the senior leading characters. They anticipated a change of appearance (e.g., appearing confident, with a more colorful outfit) to show that the character felt better after joining the program. Local male FGDs suggested categorizing the pre-intervention and post-intervention characters by the clothing color, in which a dark blue T-shirt implied sadness and loneliness and a yellow shirt showed happiness and energy. The character’s clothing can be used to distinguish the condition of the character before and after the program. Local male FGDs pointed out that one peer figure was not masculine enough and suggested to change the hairstyle and face (**Figure 7**).

##### Illustrations

**Figure 8:**
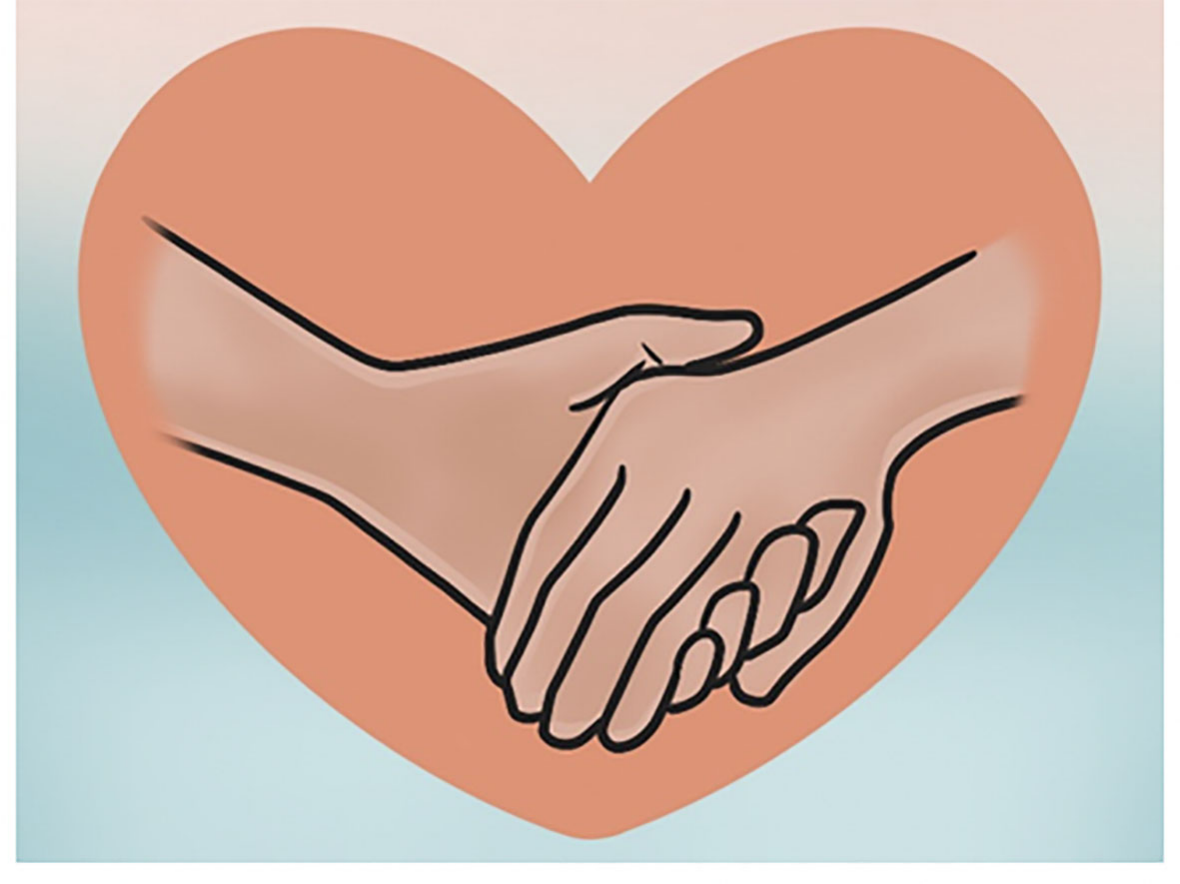
Sample illustration: “accepting help from other can make your life better”.

The illustrations were generally created based on ideas generated in FGDs. The participants decided how the illustrations looked, such as the character’s facial expression, dress, and elements of the illustration. For example, FGDs suggested an illustration of holding hand in a heart for the text of accepting help from others can make their life better (**Figure 8**).

#### Concepts

Concepts of stressors and symptoms should be consonant with the cultural and target population’s experience to make sure the terms used are relevant, acceptable, and comprehensible. Several changes were made in terms of symptoms because participants reported that the descriptions or the presence of the symptoms were not appropriate according to their experience (**Table 2**).

#### Goals

Having a congruent goal of the intervention program and the needs of clients should be consistent in digital mental health interventions. Participants in KIIs and FGDs believed that the program could be used to cope with mild and moderate mood symptoms (e.g., depression, anxiety), which is matched with the aim of the designed program. Nevertheless, experts and laypersons suggested framing the program as a stress management tool or academic performance tool instead of mental health intervention in order to reduce the stigma of using a mental health intervention, which is a common treatment barrier among Chinese young adults (42). According to KIIs, feeling stressed or complaining is a sign of incompetence among Chinese males, suggesting that this is a key element of “face” within this group. In addition, as mentioned by the local male FGDs, people who look dejected would blame themselves for being useless and not being able to change themselves or their situations. Thus, incorporating the notion of competence enhancement as one of the goals in the marketing package of the program would make the program more appealing to Chinese males.

#### Methods

Several features were designed to achieve the goals of the program within the Chinese cultural context. First, the design of the digital program allowed potential service-users to use it anytime wherever they are to reduce stigma about using mental health services. Second, although some mentioned that positive feedback in the exercise was redundant, we kept the positive feedback for all activities or adapted to a shorten version to emphasize their strength (e.g., “you did it well”), which related to the male core values and is also a motivation enhancing therapeutic strategy. Third, we adapted the planned list of activities for all groups to make the list more related to each stratum. For example, we added “go out, watch a movie” for nonlocal females, “go for a walk” for nonlocal males, “plan in some time to run/jog” for local females, and “go out with friend, go for a ride, watch Facebook video” for local males to the list of things one could do. The adapted list contained specific activities that are more appealing to one group and offered more suggested activities for potential scheduling.

#### Context

Considering context is important in the adaptation of this program to Chinese young adults because of the unique needs and experience at their developmental stage. With the partner NGO support, this program has the potential to be disseminated to most Chinese young adults and increase the accessibility of this intervention. During the FGDs, we were aware that they had concerns more than merely psychological and emotional health. They believed some of the worries were related to their academic and career development, but they had few resources for these concerns. Thus, we modified the list of local services to meet the needs of young adults, such as mental health care and career development to make the program fit the local context in this population.

## Discussion

We applied the Ecological Validity Model (26) and followed the four-phase process outlined previously (8, 31) to adapt the SbS program for Chinese young adults. We generated illustrations using a bottom-up approach, suggested as a preferable method for image adaptations in the previous adaption of SbS among Filipino migrant workers (8). Overall, we adapted the SbS to achieve acceptability, relevance, comprehensibility, and completeness of the program for the Chinese population. To our knowledge, this study is the first one to culturally adapted a digital, illustrated narrative intervention for Chinese, specifically Chinese young adults.

Several issues should be highlighted to enhance cultural adaptations. First, a proper framework and a rigorous process are essential to cultural adaptation. We employed the EVM (26) to inform cultural adaptation of SbS for a Chinese population, which allowed us to look beyond translation and to understand and collect the core cultural factors (e.g., value and spirit) to embed into the text and illustrations. Metaphors and characters reflected the epistemology and social relations within the context. Capturing the culturally sensitive elements and applying them to the mental health intervention is known to improve the impact of the intervention (43, 44). In addition, guided by a rigorous 4 stage adaptation process (8, 31), the current cultural adaptation was achieved systematically and received meaningful and valuable information from mental health experts and potential intervention beneficiaries.

Second, a successful cultural adaptation required the contribution of both experts and laypersons (8, 12). From our KIIs and FGDs, we noticed the discrepancy in preferences for leading characters between experts and laypersons. Most experts suggested senior and experienced mental health professional while FGDs favored peers and nonhuman figures. This can be interpreted in part due to the lack of professional models in the study context (45), and desire on the part of experts to put forward professional images in this program. On the other hand, the experts provided valuable information to guide us through the storyline adjustment process before FGDs. During KIIs, the experts shared their impression and experience working with Chinese young adults and suggested possible themes. It is important to balance the information and perspectives obtained from experts and service users. Feedback from the potential service users can best inform preferences for illustrations and for cultural factors, while experts are best positioned to provide suggestions related to clinical efficacy and common psychiatric problem and stressors.

Third, including layperson with proper characteristics in cultural adaptation can enhance the validity of the intervention. The current version of SbS was adapted for Chinese young adults with mood symptoms and other psychological difficulties, so incorporating participants who were Chinese young adults and with a certain level of symptoms would be beneficial to the cultural adaptation. The recruited participants in this study demonstrated minimal symptoms, since it is difficult to engage people who are actively depressed to engage in the rigorous and time-consuming cultural adaption process, so there may be a gap between what people with depression and nondepressed participants might prefer in the program and how they might perceive the text and illustrations (46). Given this concern, we focused on adaptations that are broadly relatable to the majority of the target population (e.g., emphasis their strength in encouraging messages) because they are living a similar cultural environment and sharing similar cultural experience and we believe this sufficiently informed an adequate starting point to engage the population.

Fourth, community engagement is key to a successful cultural adaptation as well as intervention implementation (47). Studies show that association involvement, as formal social ties, contributes to political trust and social trust among the participants in China (48). In this current study, we worked with the biggest local NGO that serves Chinese students. Collaboration with a local partner made the process of cultural adaptation smoother, as they assisted with providing space, access to the target population, and useful suggestions about the eventual program implementation.

Last but not least, culturally adapted interventions need to be examined. Well adapted interventions enhance the treatment effect (36). Thus, an evaluation of intervention effectiveness is necessary not only to evaluate the effect of the intervention but also to evaluate the sufficiency of the cultural adaptation. We adapted SbS for Chinese young adults, which is a culturally tailored guided self-help intervention for depression. Supported by an app-based delivery modality, the Chinese SbS is expected to be acceptable by young adults as they are generally perceived acknowledged to be proficient in digital technology. Compared with traditional face-to-face interventions, it is more accessible and promising to reduce the stigma of using a mental health intervention, which is a common barrier to mental health services among Chinese young adults (42). A majority of participants reported poor wellbeing in our study, and the adapted intervention is also possibly beneficial for the general population to learn coping skills for low mood, stress, and emotional difficulty. However, this culturally adapted version needs further evaluation (28, 29). Informed by several process models for cultural adaptation, a feasibility study is planned with a new group of Chinese adolescents, in order to test its acceptability with the target population. This study will involve cognitive interviewing to ascertain if the material requires further modification to illustrations or program narratives before testing of the effectiveness of this culturally adapted intervention in a randomized controlled trial.

### Limitation

The study has several limitations. First, the FGD participants reported minimal depressive symptoms and might perceive the program differently from those with higher levels of depression. However, we also noticed that a majority of participants from the two communities reported a low level of wellbeing, which indicates that while they did not mean cut-off scores for depression, they nevertheless reported distress. The next phase in our research process is to conduct a feasibility study with adolescents who report significant depressive symptoms. This study will provide the opportunity to evaluate how the intervention is perceived and ensure that the content is optimized for the target population.

Second, the FGD participants were mainly from three major universities/colleges out of the ten in

Macao (approximate 70% of Macau university/college students attend one of these schools) and no out-of-school young adults were included. The acceptability of the program remains unknown for students in other colleges (e.g. nursing college) and working young adults, who might have different life experiences from the FGD participants. This may affect the generalizability of the adapted materials. However, this Chinese tailored version may be applied to other Chinese young adults who study abroad and only minimal adjustment would be expected when needed, which might increase the coverage of the intervention.

Third, the stratums of local and nonlocal groups might not fully capture the variability in the sociodemographic characteristics of young adults in Macao. We also attempted to divide the focus groups into Macao local Chinese and mainland Chinese groups, since we believe that there are cultural differences between these Chinese populations, which may influence the preferred adapted content. However, we noted that there were 3 participants not born in Macao joining Macao Chinese groups, and 1 participant born in Macao joining the mainland Chinese group. The 3 Macao Chinese lived in Macao for 7, 13, and 17 years and perceived themselves as a member of the local community. However, the participant from mainland Chinese group was actually a Macao resident but perceived himself as a member of the nonlocal community. He did not consider himself a member of the local Macao Chinese community because he was raised and educated in mainland China and had lived in Macao for 5 years. This highlights the complexity of place-based identity in cultural studies.

Fourth, the size of the steering group was small. It included members with clinical psychology, communication, anthropology, and Chinese cultural background. Although the steering group did not include experts who developed the intervention, they were consulted at various stages in the adaptation process and provided additional feedback on the modifications. Guidance on the optimal number of people and types of expertise to include in the steering group is not currently available. Future studies should strive to include even more diverse perspectives, including people with expertise in human-centered design, which may complement expertise in psychology, communication, and public health for cultural adaptation studies of digital mental health programs.

Fifth, although the bottom-up approach allowed participants to generate ideas for the illustrations on their own, the participants appeared to be exhausted in the repetitive imagination process, which may reduce the efficiency of the focus group discussion. Future studies should consider including participants with proper clinical characteristics and diverse backgrounds to enhance the validity of adapted materials and the program for the target population. Using novel participatory strategies to enhance the dynamic of the discussion is recommended and might include games and mixed media interactive exercises.

### Conclusion

This study demonstrated the utility of two guidelines for cultural adaptation of SbS. It achieved a culturally appropriate version of the SbS program for Chinese young adults by adapting the elements of the program (e.g., language, metaphor, content). The current cultural adaptation study provides an example for future cultural adaptations of digital mental health interventions.

## Data Availability Statement

The raw data supporting the conclusions of this article will be made available by the authors, without undue reservation.

## Ethics Statement

The studies involving human participants were reviewed and approved by University of Macau Research Ethics Panel. The patients/participants provided their written informed consent to participate in this study.

## Author Contributions

HS managed the data, performed formal analysis, conducted investigation, and drafted the manuscript. RL assisted with investigation, analysis, and drafting paper. AL provided resourses, assisted investigation, reviewed and edited the draft. WC reviewed and edited the draft. CL provided resourses, reviewed and edited the draft. BH conceptualized the project, handled project administration, provided resourses, drafted the manuscript, and supervised the study. All authors contributed to the article and approved the submitted version.

## Funding

Funding was provided by the Macau Foundation, the University of Macau grant MYRG2018-00241-FSS, and the Johns Hopkins University Center for Global Health.

## Conflict of Interest

The authors declare that the research was conducted in the absence of any commercial or financial relationships that could be construed as a potential conflict of interest.
