# Supplementary material for: The Cultural Adaptation of Step-by-Step: An Intervention to Address Depression Among Chinese Young Adults
Source: Front Psychiatry. 2020 Jul 7;11:650. doi: 10.3389/fpsyt.2020.00650 (PMC7359726; doi:10.3389/fpsyt.2020.00650)
Supplement: Supplementary file 1 [file Table_1.docx]

Supplement 1. Interview guide of focus group discussion

|  | Contents/ Questions |
| --- | --- |
| For session 1: Warm-up | Self-introduction  Story-telling / personal experience related their difficult moment  Reflection of coping |
| For Session 1, 2, 3, 4, and 5, per segment/picture: | 1. Now I need you to help me with two things. The first is to identify the items or details in this scenario that you feel unfamiliar with, you do not understand, or make you feel uncomfortable. It could be the text, the character, even the desk, or the color. The second thing is to point out solution to modify that.  - Probe: Let’s focus on the characters and settings. What do you think about them? Can you relate with it? (e.g. appearance, experience?) What do you should be modified? - Probe: About the expression and text in this scenario, how would you change it to make it more acceptable to Chinese?  1. How about the steps to the activities? Are they easy to follow? |
| For Session 1, 2, 3, 4, and 5, after showing all segments/pictures: | 1. Now we have gone through all the pictures, what do you think happen in the story? 2. How do you think about the story in this session?  - Probe: How to make it more relatable? - Probe: How to make it more interesting or engaging?  1. Would you use this program?  - If yes, tell me why. - if not, what suggestion do you have to make it more attractive?  1. Would you recommend this to your family or friends? Why or why not? 2. What are the benefits that Chinese can gain by using this program? 3. Is the length of the session acceptable or alright with you? (i.e., too long it is boring, too short it does not provide help to you) 4. Is the pace of the session acceptable or alright with you? 5. Do you have any other thoughts about this session? |
| Review of modification from last FGD | 1. Per suggestion: In the last FGD, it was suggested to change [mention suggestion]. Were we able to make that change here? 2. Are there any additional changes that would still need to be made?  - *Probe:* Changes to make this text/picture more… - Understandable to Chinese - Relevant to Chinese - Acceptable to Chinese |
